# Electrocardiogram Changes in the Postictal Phase of Epileptic Seizure: Results from a Prospective Study

**DOI:** 10.3390/jcm12124098

**Published:** 2023-06-17

**Authors:** Lorenzo Gigli, Simone Sala, Alberto Preda, Kenji Okubo, Giovanni Peretto, Antonio Frontera, Marisa Varrenti, Matteo Baroni, Marco Carbonaro, Sara Vargiu, Chiara Di Resta, Pasquale Striano, Patrizio Mazzone, Paolo Della Bella

**Affiliations:** 1 De Gasperis Cardiocenter, Electrophisiology Unit, Niguarda Hospital, 20162 Milan, Italy; lorenzo.gigli@ospedaleniguarda.it (L.G.); marisa.varrenti@ospedaleniguarda.it (M.V.); matteo.baroni@ospedaleniguarda.it (M.B.); marco.carbonaro@ospedaleniguarda.it (M.C.); sara.vargiu@ospedaleniguarda.it (S.V.); patrizio.mazzone@ospedaleniguarda.it (P.M.); 2 Department of Cardiac Electrophysiology and Arrhythmology, IRCCS San Raffaele Hospital, Vita-Salute San Raffaele University, 20132 Milan, Italy; sala.simone@hsr.it (S.S.); peretto.giovanni@hsr.it (G.P.); dellabella.paolo@hsr.it (P.D.B.); 3 Cardiovascular Center, Yokosuka Kyosai Hospital, Yokosuka 238-8558, Japan; kenji.okubo@gmail.com; 4 IRCCS Humanitas Research Hospital, 20089 Milan, Italy; antonio.frontera@humanitas.it; 5 Faculty of Medicine, Vita-Salute San Raffaele University, 20132 Milan, Italy; diresta.chiara@hsr.it; 6 Pediatric Neurology and Muscular Diseases Unit, IRCCS Istituto Giannina Gaslini, 16147 Genoa, Italy; pasqualestriano@gaslini.org; 7 Genomic Unit for the Diagnosis of Human Pathologies, Department of Neurosciences Rehabilitation, Ophthalmology, Genetics, Maternal and Child Health (DINOGMI), University of Genoa, 16126 Genoa, Italy

**Keywords:** ECG, epilepsy, Brugada Syndrome, cardiac channelopathies, sudden cardiac death, sudden unexpected death in epilepsy

## Abstract

Background: The brain and heart are strictly linked and the electrical physiologies of these organs share common pathways and genes. Epilepsy patients have a higher prevalence of electrocardiogram (ECG) abnormalities compared to healthy people. Furthermore, the relationship between epilepsy, genetic arrhythmic diseases and sudden death is well known. The association between epilepsy and myocardial channelopathies, although already proposed, has not yet been fully demonstrated. The aim of this prospective observational study is to assess the role of the ECG after a seizure. Materials and Methods: From September 2018 to August 2019, all patients admitted to the emergency department of San Raffaele Hospital with a seizure were enrolled in the study; for each patient, neurological, cardiological and ECG data were collected. The ECG was performed at the time of the admission (post-ictal ECG) and 48 h later (basal ECG) and analyzed by two blinded expert cardiologists looking for abnormalities known to indicate channelopathies or arrhythmic cardiomyopathies. In all patients with abnormal post-ictal ECG, next generation sequencing (NGS) analysis was performed. Results: One hundred and seventeen patients were enrolled (females: 45, median age: 48 ± 12 years). There were 52 abnormal post-ictal ECGs and 28 abnormal basal ECGs. All patients with an abnormal basal ECG also had an abnormal post-ictal ECG. In abnormal post-ictal ECG, a Brugada ECG pattern (BEP) was found in eight patients (of which two had BEP type I) and confirmed in two basal ECGs (of which zero had BEP type I). An abnormal QTc interval was identified in 20 patients (17%), an early repolarization pattern was found in 4 patients (3%) and right precordial abnormalities were found in 5 patients (4%). Any kind modification of post-ictal ECG was significantly more pronounced in comparison with an ECG recorded far from the seizure (*p* = 0.003). A 10:1 higher prevalence of a BEP of any type (particularly in post-ictal ECG, *p* = 0.04) was found in our population compared to general population. In three patients with post-ictal ECG alterations diagnostic for myocardial channelopathy (BrS and ERP), not confirmed at basal ECG, a pathogenic gene variant was identified (KCNJ8, PKP2 and TRMP4). Conclusion: The 12-lead ECG after an epileptic seizure may show disease-related alterations otherwise concealed in a population at a higher incidence of sudden death and channelopathies. Post-ictal BEP incidence was higher in cases of nocturnal seizure.

## 1. Introduction

The brain and heart are strictly linked, with shared biological pathways of the electrical substrate [1]. Indeed, a number of genes code for ion channels highly expressed in both the brain and heart [2]. Consequentially, neurological diseases caused by electrical dysfunction, such as epilepsy, are usually associated with heart rhythm and repolarization abnormalities [3]. Epilepsy shares a similar age of onset with cardiac channelopathies and arrhythmic cardiomyopathies, and usually occurs between adolescence and adulthood or even earlier [4,5,6]. The type of mutation present influences the age of onset. Epilepsy patients have higher mortality compared to the general population due to an increased risk of accidental trauma, drowning, epilepticus status, suicide and also sudden death (SD), otherwise known as sudden unexpected death in epilepsy (SUDEP) [7]. Historically, the mechanism most frequently attributed to SUDEP has been asphyxia/suffocation; however, this explanation appears incomplete. Several pathophysiological conditions coexisting during the seizure could provide alternative explanations for the SD [8]. In particular, cardiac alterations such as sinus tachycardia, ictal bradycardia, or malignant arrhythmias have been described [9]. Myocardial channelopathies such as Brugada syndrome (BrS), long/short QT (LQT/SQT) and early repolarization syndrome (ERS) may cause SD. During the last few years, there has been a growing interest in exploring the possible associations between epilepsy and cardiac arrhythmias [10,11]. Among patients with BrS, there is a higher incidence of SD during sleep as well as SUDEP [12,13]. ECG screening in epilepsy patients usually reveals abnormalities in cardiac rhythm and the repolarization phase, with ST/T alterations [14]. Nevertheless, the high dynamic nature of these alterations frequently leads to misdiagnoses. Prior, during and soon after a seizure, a number of mechanisms including enhanced autonomic tone may increase ECG abnormalities and also trigger life-threatening arrhythmias [15]. Therefore, an ECG performed close to the seizure may reveal potential cardiac electrical abnormalities otherwise concealed at baseline. We previously documented a transient Brugada ECG pattern (BEP) after seizure in a woman with a rare variant of plakophilin 2 (PKP2) [16]. Another study showed a transitory shortening and prolongation of the QTc after seizure [17]. Genetic testing for cardiac channelopathies in sudden cardiac death (SCD) has developed substantially over the last few years. The next generation sequencing (NGS) technology provides an unprecedented opportunity to screen for genetic variations underlying arrhythmogenic genes in a short period of time at a low cost [18]. The aim of this observational, prospective study was to compare post-ictal ECG modifications with an ECG collected in basal conditions, focusing on seizure-related ECG variations suggestive of channelopathy or arrhythmic cardiomyopathy. The diagnostic value of post-ictal ECG was also explored using NGS analysis.

## 2. Materials and Methods

### 2.1. Patient Enrollment and Clinical Data

From September 2016 to August 2017, consecutive patients with seizure who accessed the emergency department of San Raffaele Hospital, Milan, Italy, were enrolled. In cases of out of hospital event, such as self-limited seizures, only patients evaluated within 30 min from the end of the event were enrolled. Patients were enrolled independently of the etiology of epilepsy or the type of seizure or the use of AEDs. The diagnosis of seizure was confirmed by a consultant neurologist through an integration of clinical findings (presence of tongue bite and/or post-ictal state), clinical history, a report by eventual witnesses and by using the electroencephalogram (EEG). Only the report of the EEG was collected. At the emergency department, an ECG was performed within 30 min from the end (post-ictal ECG). A basal ECG was also performed at least 48 h after the first one. All ECGs were stored in paper form and independently reviewed by two expert electrophysiologists. The research focused on electrical anomalies suggesting any channelopathy, according to universally recognized criteria [19]. The QT interval was calculated using the Bazett formula. Patients with a QTc > 470 ms and >500 ms were considered separately. Furthermore, ECG was defined as abnormal in the presence of rhythm alterations or electrical signs suggestive of right ventricular/right outflow tract conduction delay (right bundle branch block, S1S2S3 pattern, QRS fragmentation in right precordial lead). The characteristics of the seizure (onset, type, if generalized or partial, if it occurred during sleep or while awake) were collected. Anamnestic features were collected through an anamnestic interview or by consulting electronic health records. Patients with right precordial abnormalities and a negative history of arrhythmogenic cardiomyopathies underwent echocardiography and cardiac magnetic resonance (CMR) to explore the eventual presence of any structural disease. For every patient with an ECG modification suggestive of myocardial channelopathy at post-ictal ECG, a genetic analysis with next generation sequencing (NGS) was proposed. The study was conducted according to the institutional guidelines and legal requirements and it complied with the Declaration of Helsinki. All patients provided written consent for the anonymous collection of their clinical data.

### 2.2. Comparison of ECGs and Selection Criteria for ECG Changes

Post-ictal ECG and basal ECG were compared. Beyond rhythm alterations, the selected criteria to define an increase in electrical abnormality according to the ECG anomaly found were as follows:

BEP: increase in ST voltage of ST voltage ≥2 mV or prolongation of ST pattern >40 ms.
QTc prolongation: QTc increase ≥40 ms.
ERP: ST voltage increase ≥2 mV.
Right precordial abnormalities: BBD prolongation ≥40 ms, delta-wave voltage increase ≥2 mV, increase in QRS fragmentation wide ≥40 ms.

### 2.3. Blood Sample Collection, DNA Extraction and Exome Sequencing

A fresh blood sample was drawn from individual with post-ictal ECG abnormalities. Genomic DNA was extracted using a QIAamp Blood Kit (Qiagen, Hilden, Germany). A panel of 30 core genes associated with cardiac channelopathies and arrhythmogenic right ventricular cardiomyopathy (ARVC) was selected. That panel included *AKAP9*, *ANK2*, *CACNA1C*, *CACNB2*, *CASQ2*, *CAV3*, *DSC2*, *DSG2*, *DSP*, *GPD1L*, *HCN4*, *JUP*, *KNCNE1*, *KCNE2*, *KCNE3*, *KCNH2*, *KCNJ2*, *KCNJ5*, *KCNJ8, KCNQ1*, *NOS1AP*, *PKP2, RYR2*, *SCN1B*, *SCN3B*, *SCN4B*, *SCN5A*, *SNTA1*, *TMEM43* and *TMP4*. The coding exons and the flanking introns (10 bp) of each gene were amplified via a polymerase chain reaction. The pathogenicity of novel missense variants was analyzed by Alamut Visual (Interactive Biosoftware, Rouen, France) with Polymorphism Phenotyping v2 (PolyPhen-2), Sorting Intolerant from Tolerant (SIFT), MutationTaster, and Assessing Pathogenicity Probability in Arrhythmia by Integrating Statistical Evidence (APPRAISE) and that of novel splicing variants by Splice Site Finderlike, MaxEntScan, NNSPLIC, GeneSplicer and Human Splicing Finder, wherever appropriate. Splicing variants were considered to be damaging if there was a >10% lower score when compared with the wild-type prediction. Bioinformatic analyses were performed. The average sequencing coverage of the target regions was in the range of 150–250× with a 50× minimal acceptable threshold. All genetic variants detected in the index cases were validated through conventional Sanger sequencing. Variants were classified into five categories: pathogenic, likely pathogenic, variant of uncertain significance, likely benign, or benign, using the Laboratory for Molecular Medicine classification criteria. The details of the variant assessment methods and tools used have been described elsewhere [20]. All variants were evaluated for their frequency in the international population database gnomAD (http://gnomad.broadinstitute.org/, accessed on 14 November 2018); we excluded variants showing minor frequency alleles (MAF) below the calculated maximal tolerated allele frequency for the specific cardiomyopathy [21]. Polyphen2 (http://genetics.bwh.harvard.edu/pph2/; URL accessed in date 15 March 2019) and MutationTaster (http://www.mutationtaster.org/; URL accessed in date 5 April 2019) were used to predict the function of amino acid substitution. Human Splicing Finder (HSF) (http://www.umd.be/HSF/; URL accessed in date 23 September 2019) was used predict the effect of intronic variants on splicing efficiency. Moreover, bioinformatic pathogenicity evaluation for missense variants was performed by using multiple in silico tools, including Grantham distance and an Align–GVGD matrix. The splicing module from Alamut visual v.2.7.0.0 software was used to test the pathogenicity of a possible splicing variant.

### 2.4. Statistical Analysis

A structured pre-specified dataset of variables was defined and used to collect patient data. Quantitative and categorical variables were collected and analyzed according to universally recognized guidelines [22]. Continuous variables were presented as mean (standard deviation) or median (interquartile range) as appropriate, while categorical variables were presented as frequency distribution and percentage. The Shapiro–Wilk test was used to assess the normality of distribution [23]. The null hypothesis of an absence of differences between post-ictal and basal ECG was tested using a chi-square test. A level of *p* < 0.05 was chosen for statistical significance. The relationship between clinical characteristics and the presence of post-ictal ECG abnormalities was explored by performing multivariate logistic regression analysis. The odds ratio (OR) and 95% confidence interval (CI) were defined. To confirm their independent predictive value, only covariates that were significantly associated with the endpoint at univariate analysis were considered. Data were analyzed using R version 3.6.2 software (R Fundation for Statistical Computing, Vienna, Austria).

## 3. Results

### 3.1. Study Population

One hundred and seventeen patients (45 females) were enrolled. The median age was 48 (±12) years old. Sixteen patients (14%) were affected by coronary artery diseases (CAD). Structural heart disease was present in seven patients (6%). Only one patient had a positive history of SCD. The majority suffered from idiopathic epilepsy (63%). Generalized seizure was the most frequent manifestation (56%). Epileptiform modifications during EEG recording were found in 36 patients (31%). At time of admission, 40 patients were on single AED (34%) while 10 were on multiple AEDs (9%). The onset of seizure occurred during sleep in 32 patients (27%). The clinical characteristics of the study population are summarized in Table 1.

### 3.2. ECG Alterations

Overall, 80 ECGs were considered abnormal. The ECG recorded at the time of admission (post-ictal ECG) was abnormal in 52 patients (44%). BEP was found in eight post-ictal ECGs (7%), of which two were type I, two type II and four type III. A long QT interval was found in 25 patients (21%). A QTc interval greater than 470 ms was identified in 20 patients (17%), while 5 patients had a QTc > 500 ms. A short QT was found in three patients. An early repolarization pattern (ERP) was found in four patients. RV electrical disturbances were found in five patients. The basal ECG showed pathological modifications in 28 patients (24%). All patients with basal ECG alterations also have post-ictal ECG alterations. Again, QTc prolongation represented the most common abnormality: 16 patients (14%) had a long QTc, and for 14 patients it was >470 ms and for 2 patients it was >500 ms. ERP and right precordial abnormalities were noticed in four and three patients, respectively. Two cases of non-type I BEP were detected. Table 2 summarizes all ECG alterations in the population study.

### 3.3. Post-Ictal and Basal ECG Comparisons

Among the modifications described at post-ictal ECG, all BEP, seven long QTc, two short QTc and two right precordial ECG abnormalities were not confirmed on basal ECG. The QTc prolongation regression on the basal ECG observed in six patients could be explained by an electrolyte disturbance present at the time of admission and absent at the control visit, and thus this was excluded from the genetic analysis. Any kind modification in the post-ictal ECG was significantly more pronounced in comparison with the ECG recorded >48 h after the seizure (*p* = 0.003). During multivariate analysis, we found no relationship between the presence of on-going AEDs and the presence of BEPs on the post-ictal ECG at univariate analysis when adjusted for age (*p* = 0.7). However, in patients taking AEDs, we found a higher incidence of QTc prolongation (*p* = 0.04). The onset of seizure during sleep was significantly higher among patients with a BEP of any type in the post-ictal ECG (*p* = 0.04). The type of seizure did not correlate with an ECG abnormality (*p* = 0.08). All patients with electrical right precordial abnormalities had negative histories of right ventricular cardiomyopathies. The echocardiography and CMR were performed and excluded structural heart disease in all patients.

### 3.4. NGS Results

Among the 48 patients with a post-ictal abnormal ECG, 6 patients with electrolyte imbalances and 6 patients with only rhythm alterations were excluded. In total, 36 subjects underwent genetic testing using the NGS method because of a clinical suspicion of channelopathy or arrhythmic cardiomyopathy. In seven patients, inconclusive and benign variants were excluded. In two patients with a BEP I pattern, pathogenic variants of KCNJ8 and PKP2 were detected, while in one patient with ERP, a pathogenic variant of TRMP4 was detected.

## 4. Discussion

### 4.1. Main Findings

**Figure 1:**
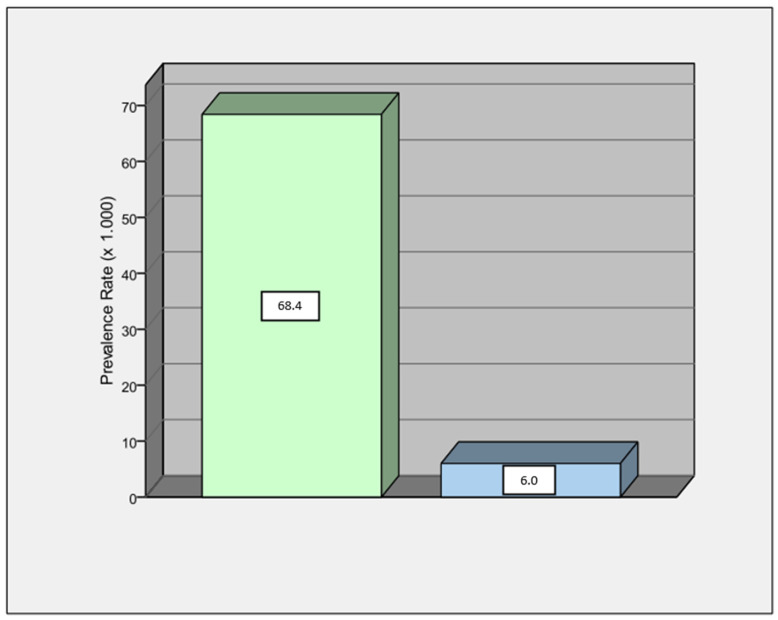
Prevalence of ECG Brugada pattern (any types) in our population (green column) appears to be around 10-fold higher in comparison to ECG Brugada pattern in general population (light blue column).

The aim of this study was to evaluate the possible diagnostic contribution provided by a post-ictal ECG in the diagnosis of electrical diseases. All patients admitted to our emergency department due to an ongoing or recent seizure were recruited. Post-ictal and basal ECGs were collected and compared. Patients with an ECG diagnosis of channelopathy or signs of possible ARVC underwent genetic analysis by NGS. The main findings of our study were as follows:

1. Patients with epilepsy have a high prevalence of ECG abnormalities, especially QTc alterations.
2. Overall, the post-ictal ECG showed more marked abnormalities than the basal ECG (*p* = 0.003).
3. The prevalence of a BEP of any type was higher in our population than in the general population with about a 10:1 ratio (Figure 1), according to a recent metanalysis [24].
4. A BEP of any type was significantly higher in the post-ictal ECG in comparison with the basal ECG (*p* = 0.04).
5. There was a relationship between seizure onset during sleep and the presence of BEP on the post-ictal ECG (*p* = 0.04).
6. In patients with ECG alterations diagnostic for myocardial channelopathy (BrS and ERP), only in early ECG, a pathogenic gene variant was identified.

**Figure 2:**
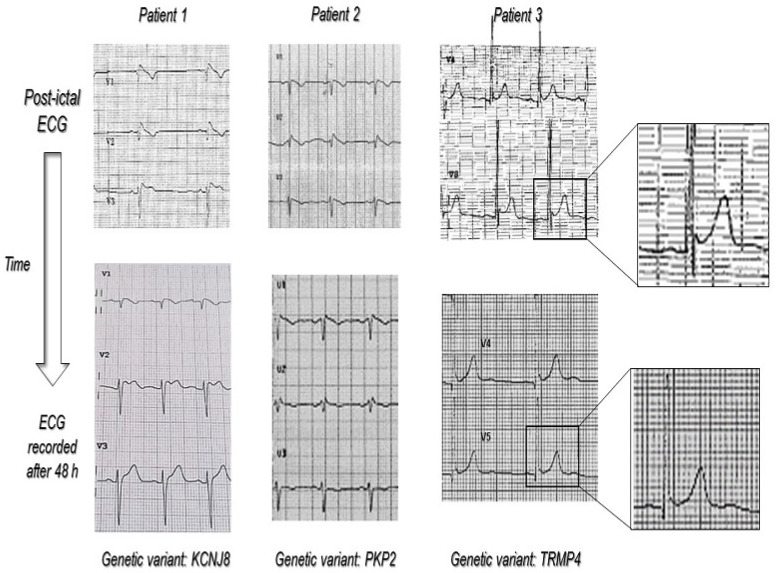
The figure shows, in detail, the ECG behavior from the post-ictal to the basal (>48 h after the seizure) time in patients with ECG modification diagnostic for myocardial channelopathy (BrS and ERS) and the respective pathogenic gene variant found using NGS analysis. The magnification in patient 3 shows how in the post-ictal ECG, the ERP was more evident. Immediately after seizure, there was a “notching” in the terminal portion of QRS and a 1 mm ST segment elevation, which disappeared in basal ECG.

The results of our study confirmed the higher incidence of ECG abnormalities in the epilepsy population. Close to the seizure time, people showed more ECG abnormalities in comparison to baseline. ECG alterations were also more marked compared to baseline in almost all patients (*p* = 0.003). We arbitrarily established thresholds to identify a significant change in the ECG, although all chosen criteria are common criteria used in clinical practice. The type of seizure has never been correlated with ECG change in previous studies and did not reach statistical significance in our study (*p* = 0.08). However, since the clinical manifestation of the seizure is usually related to the type of epilepsy, larger studies focusing on this endpoint could help to answer this question. Among abnormal ECG, the most frequent abnormality encountered was QTc prolongation. This may be mostly explained by the use of AEDs, of which QTc prolongation is quite a common side effect. Although QTc prolongation is commonly reported in patients with epilepsy, it is not clear whether it is mainly due to the use of AEDs or to the presence of a primary myocardial repolarization defect [25,26]. However, many other factors such as ion blood level or drugs interactions may influence QT interval duration. Similarly, we observed a significantly higher prevalence of a BEP of any type in comparison to the general population, with a ratio of about 10:1. BEPs were more frequent during seizures that occurred at night. This finding is in agreement with the well-known higher incidence of BrS-related ventricular arrhythmias at nighttime [27]. According to the most recent guidelines, only BEP type I can be considered diagnostic and deserves further investigations [19]. In our study, all diagnostic BEPs present in the post-ictal ECG disappeared in the basal ECG. A number of genes have been linked to genetic cardiomyopathies with a high risk of SCD [28]. The screening of these genes individually using traditional Sanger sequencing is labor-intensive and expensive. High-throughput, cost-effective genetic testing methods are urgently needed. The NGS approach provides opportunities to identify and investigate thousands of genetic aberrations simultaneously among diverse cardiomyopathy phenotypes, and it is an important auxiliary tool for clinicians regarding decision-making, diagnosis and treatment. In this study, the genetic analysis identified the pathogenic variant of ion channels in patients with relevant ECG modifications only in the post-ictal phase (Figure 2). Indeed, in three cases with a non-diagnostic basal ECG, a pathogenic variant was identified. In those three cases, we can conclude that the sensitivity of the post-ictal ECG was higher than the basal ECG. However, further studies are needed to confirm this hypothesis.

### 4.2. Arrhythmogenic Diseases in Patients with Epilepsy

Patients with epilepsy are more likely to have abnormal QTc intervals, ST segment abnormalities, elevated T waves, ERP, increased *p* wave dispersion and PR intervals when compared to healthy people [14,29]. In the majority of cases, pathological ECG findings has been observed during the repolarization period with a transient QTc interval and ST tract modifications [30]. Moreover, a wide spectrum of ECG alterations has been described before, during and after a seizure [31,32]. During a seizure, cardiovascular and cardiorespiratory reflexes associated with the side effects of some AEDs contribute to ictal cardiac changes. Of note, cardiac manifestations often precede the onset of seizure. Both cardiac autonomic impairment and conduction system dysfunction are well reported in patients with epilepsy [33,34]. Changes in vagus nerve activity (which have a great influence on the heart) could predict seizure onset [3]. Changes in heart rate are the most common findings but rarely manifest together with life-threatening brady- or tachyarrhythmias [35,36]. However, a few studies explored the possible diagnostic value of post-ictal ECG in detecting genetically determined arrhythmic diseases [29]. Electrical changes in post-ictal ECG were well described only in epilepsy patients affected by a structural brain disease [37]. No studies investigated patients with these high-risk ECG abnormalities using a genetic assessment.

The results of our study may support the hypothesis that among epileptic patients, the prevalence of myocardial channelopathies may be higher [38]. Indeed, it is well known that channelopathies and epileptic syndromes share common genes [39,40]. Despite the fact that the most common electrical alterations during seizure concern heart rhythm [31,41], repolarization abnormalities diagnostic for channelopathies, such as BrS, long QT syndrome and ERP, in epilepsy patients are largely reported in the literature [25,42], including their dynamic ECG nature. On the contrary, the link between ERP and epilepsy is less clear. Some studies reported a higher abnormal ER in epilepsy patients than in controls, independently of the type of epilepsy [38,43]. However, few data are available so far, probably due to several pitfalls in the ECG diagnosis, the high prevalence of benign repolarization patterns in the general population, low prevalence of ERP and the lack of knowledge on its pathophysiology. In one ERP patient, a pathological mutation in TRPM4 was revealed. According to several studies, the TRPM family is largely involved in the genesis of epilepsy [44]. Electrical right precordial abnormalities were all judged to be non-specific after additional analysis. Indeed, ECG alterations alone are not sufficient criteria of ARVC and its coexistence with epilepsy is rare [45]. The most severe manifestation of “brain-heart” link impairment is SUDEP, which provides the greatest contribution to epilepsy-related mortality accounting for an increased risk of sudden death in chronic epilepsy of 20–40 times compared to the general population [34]. SUDEP occurs more often in people from 21 to 40 years old, and therefore in a slightly younger age than the mean age of our population [46]. Cardiac arrhythmias are considered to have a fundamental role in SUDEP and tachycardia, bradycardia, supraventricular tachyarrhythmias, LQT syndrome or SQT syndrome [17,47,48,49,50,51,52,53,54,55]. The possibility of an arrhythmogenic etiology of SUDEP due to an unknown concomitant myocardial channelopathy has been considered and several data support the hypothesis of this correlation. The NGS era has led to the identification of many ion channel mutations, both in epileptic patients and patients with myocardial channelopathies, offering a deeper view of a possible correlation between myocardial and neurological syndromes [56]. In generalized epilepsy with febrile seizures plus (GEFS +) the presence of a gene SCN1B mutation (sodium channel voltage-dependent encoding gene, type I, β subunit) was reported, also described in the BrS [57]. In a recent study, SCN5A and KCNH2 mutations (voltage-dependent potassium channel encoding gene, subfamily H, member 2) were identified in more than 10% of subjects who died of SUDEP [58]. A SUDEP case has been reported in a patient with idiopathic epilepsy and SCN5A gene mutations [59]. The same SCN5A gene point mutation was isolated in three consanguineous subjects with BrS and epilepsy [11].

### 4.3. The Integrative Role of Post-Ictal ECG in Patients with Epilepsy

**Figure 3:**
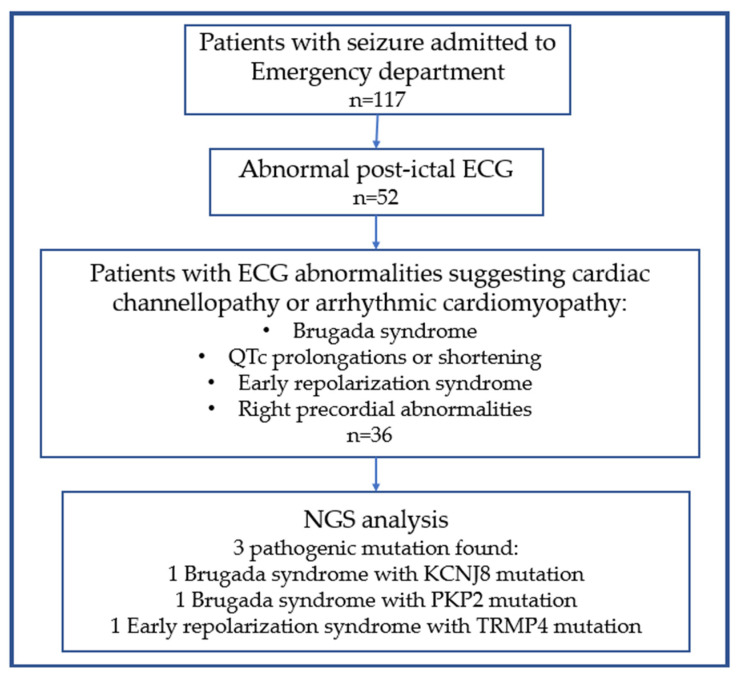
Operative diagnostic workflow.

Our study showed, despite the limited sample size, that comparing post-ictal and basal ECG in this type of population could help to identify patients with arrhythmogenic diseases at an increased risk of SCD. Figure 3 summarizes the operative flow-chart that we followed. The sensitivity of a resting ECG in the diagnosis of cardiovascular disease, including cardiac channelopathies or cardiomyopathies, is generally scarce. This method has not evolved over the decades. The diagnosis requires a high level of suspicion because the resting ECG is often borderline, intermittently normal, or frankly normal. Nevertheless, it still remains the most used in clinical practice for both screening and diagnosis as it is widespread and cost-saving. Genetic testing does not provide a simple solution to this issue, as it is often neither sensitive nor specific, even when the phenotype points to a specific entity and may yield results that are difficult to interpret (i.e., variants of unknown significance). Pharmacological and/or exercise testing by manipulation/stressing the cardiac action potential can accentuate the desired abnormality or provoke a characteristic arrhythmia response. In this context, a seizure might be considered as a stress test to improve ECG sensitivity, and, maybe, specificity [60]. Unlike arrhythmogenic cardiomyopathies such as ARVC, in which the progression of structural ventricular alterations can be preceded and predicted by ECG depolarization/repolarization abnormalities (despite the low diagnostic specificity) [61], in cardiac channelopathies, structural alterations appear very late or not at all. Therefore, a greater importance is given to correct ECG interpretation (usually by consulting a specialist electrophysiologist) and to anamnestic interview [62]. Since the first manifestation of the disease can be SCD, the primary challenge in SCD prevention is the early identification of individuals at risk. Performing an ECG during or soon after a seizure could help to select patients that deserve further investigations. Currently, the role of ECG in the diagnosis and management of both epilepsy and seizures is minor compared to EEG, although it has been shown to be useful in screening [63], home monitoring [64] and, in some cases, when there is a false negative EEG [65]. In one study, the ECG predicted ictal phases [66]. Another study reported a higher incidence of new ECG alterations being related to a higher risk of SCD in patients with refractory epilepsy compared to a drug-sensitive one [67]. Only few studies reported abnormal EEG findings in patients with BrS and LQT syndrome [68,69], but no studies aimed to explore EEG/ECG variations in the different types of epilepsy as well as the possible presence of EEG abnormalities in other channelopathies or genetic cardiomyopathies.

## 5. Limitations

This is an observational, single-center prospective study. Therefore, it has the inherent limit of study design and thus our results must be confirmed in a larger sample. In this study, we reported our experience in identifying ECG changes in patients with epilepsy through a comparation of post-ictal and basal ECGs. Since channelopathies are relatively rare diseases, a larger sample is needed to provide significant correlations among the variables in the study and the differences in early and late ECGs for all detected anomalies. The absence of follow-up did not permit us to answer the following question: do patients with an abnormal post-ictal ECG have an increased risk of SUDEP? Furthermore, ECG alterations associated with myocardial channelopathies are often fluctuant and so it is difficult to demonstrate a cause–effect relationship between seizures and “unmasked” alterations. Finally, EEG reports were not recorded, thus not allowing comparisons with ECGs.

## 6. Conclusions

This single-center observational prospective study showed that a 12-lead ECG performed immediately after a seizure may show disease-related alterations that are otherwise concealed in a population at higher incidence of SD and channelopathies. Epilepsy patients reported a higher prevalence of BEPs of any type compared to healthy individuals. A relationship between nocturnal seizures and post-ictal BEP was found.

## Figures and Tables

**Table 1:** Clinical characteristics of the study population.

Variable	Total
No. of patients	117
Age, median y (±IQR)	48 ± 12
Male (%)	72 (62%)
Neurological characteristics	
Etiology of epilepsy (%)	
Idiopathic	74 (63%)
Genetic	1 (1%)
Structural	42 (36%)
Neoplastic	14 (12%)
Vascular	23 (20%)
Dysplasia	4 (4%)
Type of seizure (%)	
Generalized	65 (56%)
Focal	19 (16%)
Unknown	33 (28%)
Seizure during sleep (%)	
Yes	32 (27%)
No	86 (74%)
EEG at time of admission (%)	
Not IEDs	81 (69%)
IEDs	36 (31%)
Ongoing AEDs	
Single	40 (34%)
Multiple	10 (9%)
None	67 (57%)
Cardiological characteristics	
Hypertension	33 (28%)
Diabetes Mellitus	18 (15%)
CKD	7 (6%)
CAD	16 (14%)
SCD family history	1 (1%)
Previous arrhythmias	5 (4%)
Structural heart disease	7 (6%)

AEDs, antiepileptic drugs; CAD, coronary artery disease; CKD, chronic kidney disease; EEG, electroencephalogram; IEDs, interictal epileptiform discharges; SCD, sudden cardiac death.

**Table 2:** ECG features of the study population.

Variables	Total (%)
Post-ictal ECG abnormalities	52 (44%)
Rhythm disturbances	
Tachyarrhythmias	3 (3%)
Bradyarrhythmias	3 (3%)
Brugada pattern type I	2 (2%)
Brugada pattern type II–III	6 (4%)
QTc modifications	
QTc prolongation (>470 ms)	20 (17%)
QTc prolongation (>500 ms)	5 (4%)
QTc shortening (<340 ms)	3 (3%)
ERP	4 (3%)
Right precordial abnormalities	5 (4%)
Basal ECG abnormalities	28 (24%)
Rhythm disturbances	
Tachyarrhythmias	2 (2%)
Bradyarrhythmias	0
Brugada pattern type I	0
Brugada pattern type II–III	2
QTc modifications	
QTc prolongation (>470 ms)	14 (12%)
QTc prolongation (>500 ms)	2 (2%)
QTc shortening (<340 ms)	1 (1%)
ERP	4 (3%)
Right precordial abnormalities	3 (3%)

ECG, electrocardiogram; QTc, corrected QT interval; ERP, early repolarization.

## Data Availability

At the request of the editor.
